# Antioxidants and Cardiovascular Risk Factors

**DOI:** 10.3390/diseases4010011

**Published:** 2016-02-17

**Authors:** Daniela Pellegrino

**Affiliations:** Department of Biology, Ecology and Earth Sciences (DiBEST), University of Calabria, 87036 Italy; danielapellegrino@unical.it; Tel.: +39-0984-492-924

**Keywords:** cardiovascular disease, antioxidants, oxidative stress

## Abstract

Cardiovascular disease (CVD), the world’s primary cause of death and disability, represents a global health problem and involves a great public financial commitment in terms of both inability to work and pharmaceutical costs. CVD is characterized by a cluster of disorders, associated with complex interactions between multiple risk factors. The early identification of high cardiovascular risk subjects is one of the main targets of primary prevention in order to reduce the adverse impact of modifiable factors, from lifestyle changes to pharmacological treatments. The cardioprotective effect of food antioxidants is well known. Indeed, a diet rich in fruits and vegetables results in an increase in serum antioxidant capacity and a decrease in oxidative stress. In contrast, studies on antioxidant supplementation, even those that are numerically significant, have revealed no clear benefit in prevention and therapy of CVD. Both short- and long-term clinical trials have failed to consistently support cardioprotective effects of supplemental antioxidant intake. The aim of this review is to evaluate the antioxidant effects on the main cardiovascular risk factors including hypertension, dyslipidemia, diabetes.

## 1. Introduction

Cardiovascular disease (CVD) is the principal cause of death and disability in the developed countries and the most important cause of premature death worldwide, as outlined by the World Health Organization [1]. In the USA, an estimated one in three adults presents atherosclerotic vascular disease, and the global absolute risk of experiencing a major cardiovascular event after age 50 is about 52% for men and 39% for women [2,3]. Thus, CVD represents a global health problem for healthcare systems in terms of both inability to work and pharmaceutical charges, and strategies to prevent CVD have universal significance on health outcomes and healthcare expenditures. Pathologies that may affect the heart and the blood vessels include hypertension, coronary heart disease, cerebrovascular disease, peripheral vascular disease, heart failure, cardiomyopathies, and rheumatic and congenital heart disease. Indeed, CVD is characterized by a group of disorders, related to intricate interactions among risk factors.

Cardiovascular prevention seen as risk factor identification has a long and successful history. In 1961, the Framingham study identified a series of risk factors with pathogenic implications for CVD in subjects aged between 40 and 69 years [4]. The main risk factors included smoking, hypertension, dyslipidemia, and diabetes [5]. Further important predisposing factors included high fat diet, low physical activity, obesity, and genetic influences [5]. Over the years, scientific evidence has shown that in the case of CVD, an effective prevention is possible and useful even from the economic viewpoint [6]. The CVD prediction models based on the Framingham study, still used all over the world, have been confirmed by several epidemiological studies and have contributed to a stable reduction in CVD mortality [7]. In recent years, attention was focused toward seeking new biomarkers with no direct pathogenic effect but with a verified association with CVD in order to target preventive treatments of asymptomatic subjects [8,9].

It has long been known that an alteration in oxidative status is associated with many chronic diseases such as neurodegenerative conditions, inflammatory diseases, cancer and CVD [10,11,12]. Oxidative stress includes any condition in which oxidant metabolites can exert their damaging effects because of increased production or altered cellular mechanisms of protection. Reactive oxygen species (ROS), generated at the vascular endothelium level, act as a significant factor in endothelial dysfunction underlying the development of atherosclerosis and chronic kidney disease (CKD) [13,14]. CKD patients show high levels of oxidative stress and inflammation [14] closely associated with an impaired mitochondrial respiratory system [15] and this condition may be both the consequence and the cause of an enhanced oxidative stress. Furthermore, it has been shown that lower total antioxidant response and higher total peroxide levels are associated with cardiomyopathy, atherosclerosis and coronary artery disease [16,17,18,19,20].

Although the ability of oxidative stress biomarkers to predict CVD has yet to be established [21], the cardioprotective effect of food antioxidants has long been known. In humans, a diet based on antioxidant-rich foods results in an increase in serum antioxidant capacity and a decrease in oxidative stress [22,23,24], and protects from cancer and heart disease [25]. Administration of antioxidants as dietary supplements does not seem to have the same beneficial effect [26]. Several clinical trials found that antioxidant supplementation does not reduce CVD risk, and, in some cases, appears to increase the risk of some types of cancer [25]. For these reasons, and also to limit the immoderate consumption of antioxidant supplements, both the US Food and Drug Administration and the European Food Safety Authority have excluded any information that could imply potential health benefits of products with antioxidants from the package labels [27,28].

The main focus of this review is to analyze the effects of antioxidant-rich food and antioxidant supplementation on traditional cardiovascular risk factors such as hypertension, dyslipidemia, and diabetes.

## 2. Hypertension

Several studies provide strong evidence for a sustaining role of oxidative stress in hypertension. In the vessel wall, all cell types produce ROS in variable quantities and in response to different stimuli, which may act in an autocrine or paracrine way to modulate the cellular function [29,30]. In experimental models of a hypertensive rat, an increased blood pressure is associated with increased oxidative stress [31]. Oxidative stress experimentally induced with lead initiates hypertension in both cultured endothelial cells and intact animals [32,33,34], as well as benefits from antioxidants treatment [35]. Furthermore, hypertension is mediated by increased oxidative stress resulting from the experimental altered antioxidant capacity (glutathione synthase inhibition, [36]; superoxide dismutase inhibition, [37,38]). In human trials, it has been shown that superoxide dismutase enzymatic activity is remarkably reduced in hypertensive patients when compared with normotensive controls [39]. Moreover, it has been demonstrated that there is a decrease in the endogenous antioxidant enzyme levels and an increase in lipid peroxidation markers in hypertensive subjects; in these patients, treatment with antihypertensive drugs showed a two-fold benefit: a decrease of blood pressure and an overall improvement in the oxidative stress [40]. Collectively, these data indicate that oxidative stress plays a critical role in regulating the long-term set point of arterial pressure but that which has not been sufficiently clarified is whether oxidative stress is an effect or a cause of essential hypertension [41].

An antioxidant molecule highly studied for its antihypertensive effect is the natural polyphenolic compound resveratrol. Already in 1992 Renaud and De Lorgeril assumed that the regular consumption of red wine, particularly rich in resveratrol, was an important factor of the “French Paradox”, the term used to describe the low incidence of CVD in the Southwestern French population despite its high intake of saturated fats [42]. An important cardioprotective property of resveratrol is linked to oxidative stress. In fact, resveratrol treatment reduces ROS production and up-regulates the endogenous antioxidant systems in both endothelial and cardiac cells [43,44]. Resveratrol treatment also prevents platelet activation by modulating platelet adhesion, secretion and activation signaling, ROS production, and apoptosis and by enhancing nitric oxide (NO) production [45]. The antihypertensive effect of resveratrol dietary supplements has been demonstrated in several hypertensive animal models and an improvement of endothelial function (*i.e.*, endothelial NO synthase activation) was also described [26]. Unlike the preclinical studies, limited and conflicting information is available regarding the antihypertensive effect of resveratrol supplementation in humans. A meta-analysis of randomized controlled trials has shown that ingestion of resveratrol (both at high and at low doses) decreases systolic blood pressure without affecting diastolic blood pressure [46]. Other studies have shown that resveratrol supplementation did not exert any effect on blood pressure in healthy obese adults, in patients with metabolic syndrome and in patients with previous myocardial infarction [26]. Besides, a significant aspect to be considered in the antioxidant supplementation is the pharmacokinetics of the substance and, hence, its bioavailability. The consumption of red wine leads to a highly efficient resveratrol uptake while resveratrol supplementation leads to a very low bioavailability. The reason for this appears to be the requirement for a particular matrix, such as the phytocomplex of wine, in which resveratrol works in synergy with other compounds [47].

The antioxidant vitamins C and E have been tested as therapeutic agents for hypertension as both vitamins down-regulate NADPH oxidase (nicotinamide adenine dinucleotide phosphate-oxidase, a major ROS source in the vascular wall) and up-regulate endothelial NO synthase (eNOS), resulting in a blood pressure lowering effect [48,49]. Despite a number of studies that have shown the antihypertensive effects of these vitamins, the majority of large clinical trials did not find any clear benefit after antioxidant supplementation in hypertension treatment and cardiovascular mortality [50]. On the contrary, antioxidant and antihypertensive effects of a diet rich in fruits and vegetables have been reported in both healthy subjects and patients with hypertension [50].

## 3. Dyslipidemia

Dyslipidemia is characterized by an elevated fasting and postprandial plasmatic concentration of total triglycerides and free fatty acids, associated with high levels of low-density lipoproteins (LDL) cholesterol and low levels of high-density lipoproteins (HDL) cholesterol. This lipid profile in combination with endothelial damage is a crucial event in the most common pathological processes underlying CVD [51,52].

HDL cholesterol represents a key cardioprotective factor given its role in reverse cholesterol transport, its effects on endothelial cells, and its antioxidant activity [53]. On the other hand, hypertriglyceridemia leads to an increase of small dense LDL by metabolization delay and consequent atherogenicity [54]. In addition, endothelial dysfunction can be started/supported by several factors, including an excess of ROS and the exposure to harmful agents such as oxidized LDL [55].

Disorders of lipid metabolism are associated with the overproduction of reactive oxygen species and have been shown to affect the antioxidant status of different organs as well as their lipoprotein levels [56]. The common side effects of the current synthetic lipid-lowering drugs [57] have increased the tendency to move toward traditional and alternative treatments for the prevention of hyperlipidemia including medicinal plants and natural antioxidants. Lipid-lowering compounds, available in food supplements and medicinal plants, can be effective for the metabolism of lipids by influencing the metabolic reactions of different tissues and, in most cases, these lipid-lowering properties, at least in part, have been attributed to their antioxidant properties [58]. Literature data on the effects of foods and bioactive compounds showed that several nutrients and food components could positively impact the lipid profile (monounsaturated and polyunsaturated fatty acids, soluble fiber, vegetable proteins, phytosterols, and polyphenols; [59]). A high intake of vegetables and fruits was associated with better control and regulation of the lipid profile [60]. Furthermore, regular consumption of oleic acid decreased LDL cholesterol without lowering HDL cholesterol [61]. In contrast, supplementation of the isolated bioactive compounds showed contradictory effects. For example, supplementation with the flavonoid pigment anthocyanin exerted beneficial metabolic effects in Chinese subjects with type 2 diabetes by improving dyslipidemia, enhancing antioxidant capacity, and preventing insulin resistance [62]. Conversely, a meta-analysis of randomized controlled trials showed that resveratrol supplementation had no significant effect on plasma concentrations of total cholesterol, LDL cholesterol, HDL cholesterol, and triglycerides [63]. However, this meta-analysis examined studies with heterogeneous population characteristics, including normolipidemic subjects and hyperlipidemic patients on statin therapy. Hence, any hypocholesterolemic effect of resveratrol may be masked by the potent effects of statin therapy.

## 4. Diabetes

Diabetes mellitus is a multifactorial disease characterized by a high value of plasma glucose due to either pancreatic failure to secrete insulin (type 1) or cellular insulin resistance (type 2). Diabetes is a significant cardiovascular risk factor and, at the same time, CVD is the major cause of morbidity and mortality in type 1 diabetes mellitus [64]. There is increasing evidence for the role of oxidative stress in the development of both types of diabetes, although the causative role of redox imbalance in the development and/or progression of the disease remains unclear [65]. Oxidative stress would seem to be a key factor in diabetic complications, including CVD, and it is also closely associated with insulin resistance and impaired insulin secretion, resulting in the development of the disease [66]. Cellular models suggest that pancreatic β-cells are particularly vulnerable to oxidative stress and insulin gene expression is down-regulated in isolated rat β-cells exposed to free radicals [67]. Moreover, in diabetic mice, antioxidant treatment improved glucose-stimulated insulin secretion with a consequent decrease in blood glucose [67]. The increased ROS production in diabetes is due to different mechanisms including increased mitochondrial ROS production and increased activity and expression of NADPH oxidase [66]. Increased ROS production was often observed in obesity and is closely linked to the development of metabolic syndrome and diabetes [68]. Clinical studies have also shown that antioxidant concentrations in plasma and erythrocytes of diabetes subjects are reduced [69]. Both *in vivo* and *in vitro*, ROS have proven to influence antioxidant action in a diabetic state: in the kidneys of diabetic mice, the superoxide dismutase (SOD) expression and the total SOD activity has been shown to be decreased; in diabetic mouse models, SOD expression is reduced in multiple tissues, including heart, brain, kidney and liver [66]. It should be noted that diabetes is often accompanied by micronutrient deficiencies including antioxidants such as vitamin D, whose antioxidant property is rather newly recognized and less studied. In experimental models, vitamin D supplementation inhibits lipid peroxidation but did not carry additional benefit over insulin injection alone to decrease oxidative stress [70] while its beneficial effects on glycemic control have been shown in some recent clinical studies [65]. Clinical findings on antioxidant functions of vitamin D in diabetes are scarce [71], though promising results emerged from a study on the antioxidant effects of daily supplementation with fruits in diabetic subjects [72]. Recent meta-analyses of studies examining the effect of vitamin D on type 2 diabetes and cardiovascular disease risk factors have produced largely negative results while some studies have suggested that improvements are only observed in vitamin D-deficient individuals and only with adequate vitamin D supplementation [73]. Furthermore, the side effects of long-term vitamin D administration including the hypercalcemia and hyperphosphatemia, which could potentially lead to kidney nephrolithiasis, should be considered [74]. Although several experimental data support the hypothesis of an active involvement of oxidative stress in diabetes development, the human studies are currently insufficient to support an effectiveness of antioxidant supplementation.

## 5. Conclusions

The current literature data highlight that a diet rich in antioxidants is able to positively modulate the main cardiovascular risk factors thought to enforce the organism’s ability to counteract free radicals. Conversely, antioxidant administration in the form of dietary supplements does not seem to have the same beneficial effect. The conflicting data that emerged from preclinical and clinical studies emphasizes the importance of evaluating the antioxidant mixtures, such as those found in natural products, rather than simple antioxidant formulas, due to synergism between antioxidants. Furthermore, it should be taken into account that (i) administration of exogenous antioxidants may inhibit the endogenous synthesis; (ii) antioxidant effects may be due to indirect effects (induction of endogenous antioxidant mechanisms); (iii) antioxidant substances may have a plethora of other effects *in vivo*, not related at all to its antioxidant action; (iv) antioxidant effects may be strongly influenced by factors such as population characteristics, basic oxidative status and medical conditions and comorbidities.

In addition, future studies on antioxidants should take into great consideration their biochemical characteristics, *i.e.*, water (hydrophilic)- or lipid (hydrophobic)-soluble substances. Most water-soluble antioxidants are distributed only in the cytosol while hydrophobic substances are able to easily cross both cell membranes and the mitochondrial membrane and accumulate in mitochondria which represents an important site of ROS production involved in CVDs. For instance, mitochondrion-permeable antioxidants (Edaravone, idebenone, 𝛼-Lipoic acid, carotenoids, vitamin E, coenzyme Q10) and mitochondria-targeted antioxidants (MitoQ and SkQ) should be much more effective than water-soluble antioxidants [75].

In conclusion, several data support the hypothesis of an active involvement of oxidative stress in the main cardiovascular risk factors. Although the human studies are currently insufficient to support the use of antioxidant supplementation as a preventive and/or therapeutic agent, results obtained to date are promising. Further studies are required to better understand the complex oxidative equilibrium under physiological/pathological conditions.
